# Supplementary material for: Neural oscillatory dynamics reveal altered top-down and integrative mechanisms during face processing in autistic children and unaffected siblings of autistic children
Source: J Neurodev Disord. 2026 May 8;18:38. doi: 10.1186/s11689-026-09706-z (PMC13326095; doi:10.1186/s11689-026-09706-z)
Supplement: Supplementary file 1 — Supplementary Material 1. Supplementary figure 1. (A) Topographical representation of the difference wave (Upright Faces – Upright Objects) at the timing of the main components: P1 (135 ms), N170 (180 ms) and the P2 (240 ms) for the NA group. (B) Difference wave for P7 (left) and P8 (right) for the NA group. Time-points that are significantly different than 0 are highlighted in grey rectangles or a green rectangle to illustrate significant differences at the timing of the N170. (C) Spatio-temporal cluster plot of one-sample t-test performed for each time-point and each channel with significant spatio-temporal cluster highlighted by a black outline. (D) and (E) same structure but for AU and SIB respectively. (F) Difference wave (Upright Objects – Inverted Objects) for NA (left), AU (middle) and SIB (right) groups, for P7 (top row) and P8 (bottom row). Supplementary figure 2. Similar alpha event-related desynchronization between groups and conditions. (A) Topographical representation of the induced alpha activity (7 – 13Hz) averaged between 200 to 500 ms in response to each type of stimulation and (B) time course of induced alpha power change (in percent change from baseline) averaged over a cluster of occipital channels in response to Upright Faces (red), Inverted Faces (orange), Upright Objetcs (blue) and Inverted Objects (light blue), for NA (left), AU (middle) and SIB (right)., for NA (left), AU (middle) and SIB (right). Supplementary figure 3. Similar beta event-related desynchronization between groups and conditions. Top-panel: Time-frequency representations of the induced spectral activity (4 – 40Hz) averaged over a cluster of occipital channels to each type of stimulation (columns) and for each group (rows). Bottom-panel: Time course of induced beta power change (in percent change from baseline) averaged over a cluster of occipital channels in response to Upright Faces (red), Inverted Faces (orange), Upright Objetcs (blue) and Inverted Objects (light blue), fo [file 11689_2026_9706_MOESM1_ESM.docx]

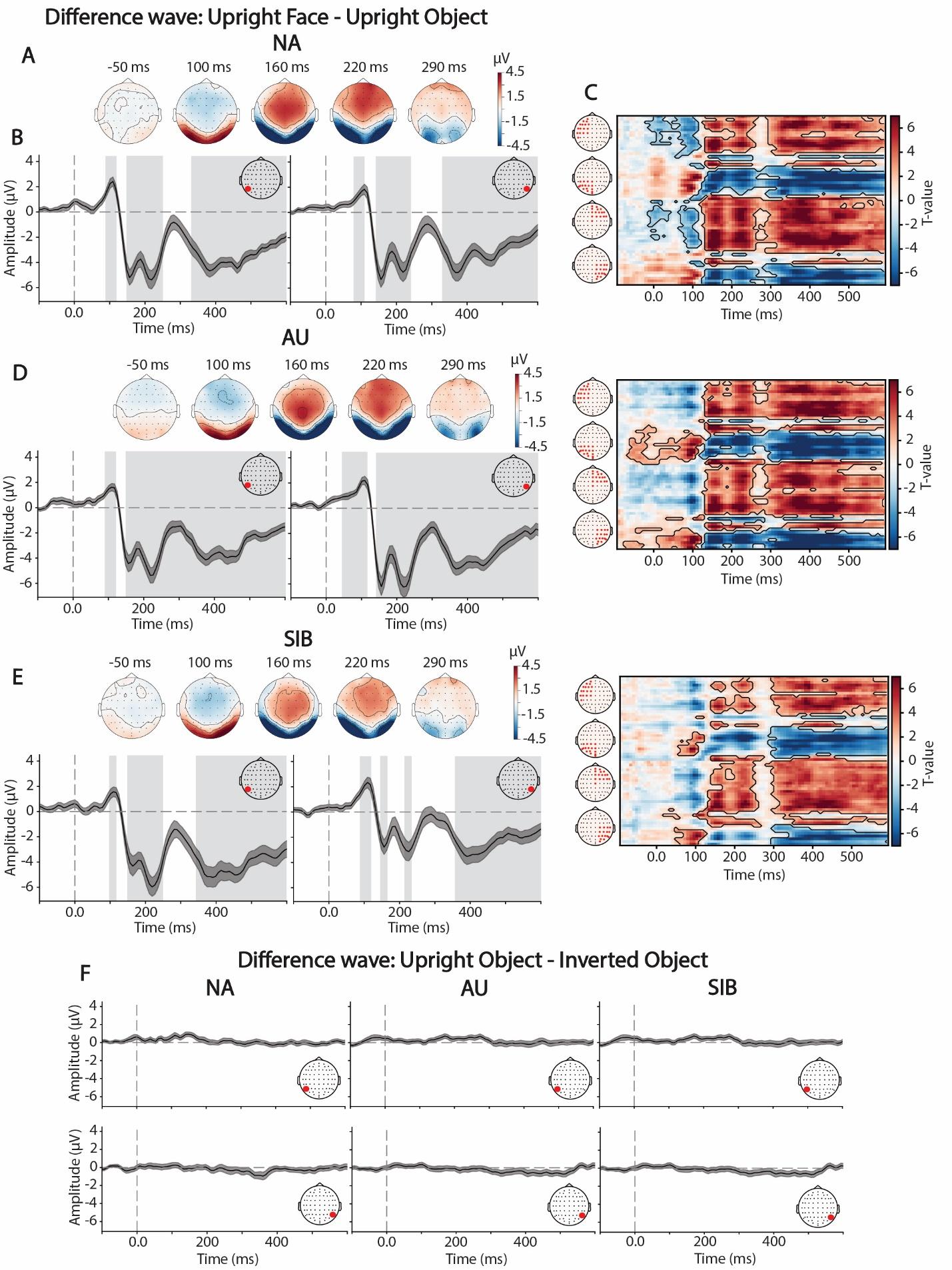


**Supplementary figure 1. (A)** Topographical representation of the difference wave (Upright Faces – Upright Objects) at the timing of the main components: P1 (135 ms), N170 (180 ms) and the P2 (240 ms) for the NA group. **(B)** Difference wave for P7 (left) and P8 (right) for the NA group. Time-points that are significantly different than 0 are highlighted in grey rectangles or a green rectangle to illustrate significant differences at the timing of the N170. **(C)** Spatio-temporal cluster plot of one-sample t-test performed for each time-point and each channel with significant spatio-temporal cluster highlighted by a black outline. **(D)** and **(E)** same structure but for AU and SIB respectively. (F) Difference wave (Upright Objects – Inverted Objects) for NA (left), AU (middle) and SIB (right) groups, for P7 (top row) and P8 (bottom row).

**
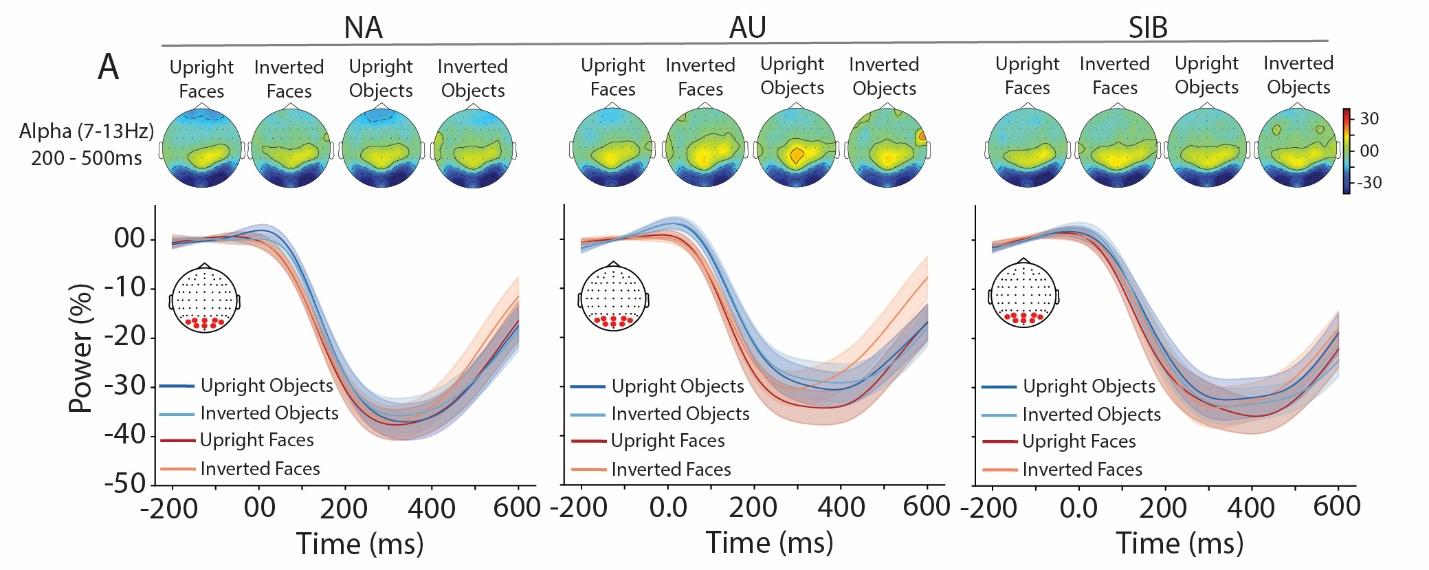
**

**Supplementary figure 2. Similar alpha event-related desynchronization between groups and conditions. (A)** Topographical representation of the induced alpha activity (7 – 13Hz) averaged between 200 to 500 ms in response to each type of stimulation and **(B)** time course of induced alpha power change (in percent change from baseline) averaged over a cluster of occipital channels in response to Upright Faces (red), Inverted Faces (orange), Upright Objetcs (blue) and Inverted Objects (light blue), for NA (left), AU (middle) and SIB (right)., for NA (left), AU (middle) and SIB (right).


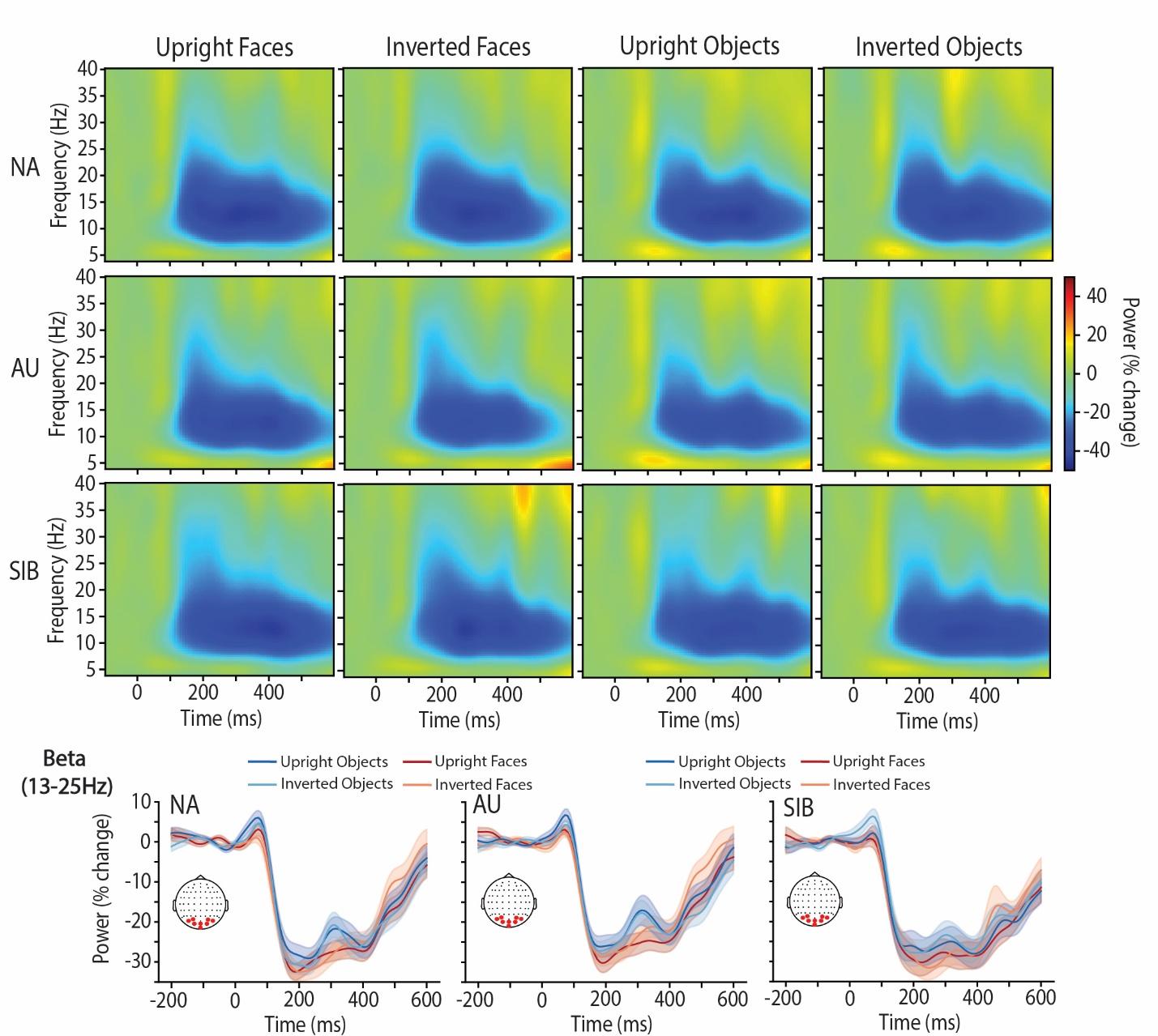


**Supplementary figure 3. Similar beta event-related desynchronization between groups and conditions. Top-panel:** Time-frequency representations of the induced spectral activity (4 – 40Hz) averaged over a cluster of occipital channels to each type of stimulation (columns) and for each group (rows). **Bottom-panel:** Time course of induced beta power change (in percent change from baseline) averaged over a cluster of occipital channels in response to Upright Faces (red), Inverted Faces (orange), Upright Objetcs (blue) and Inverted Objects (light blue), for NA (left), AU (middle) and SIB (right).


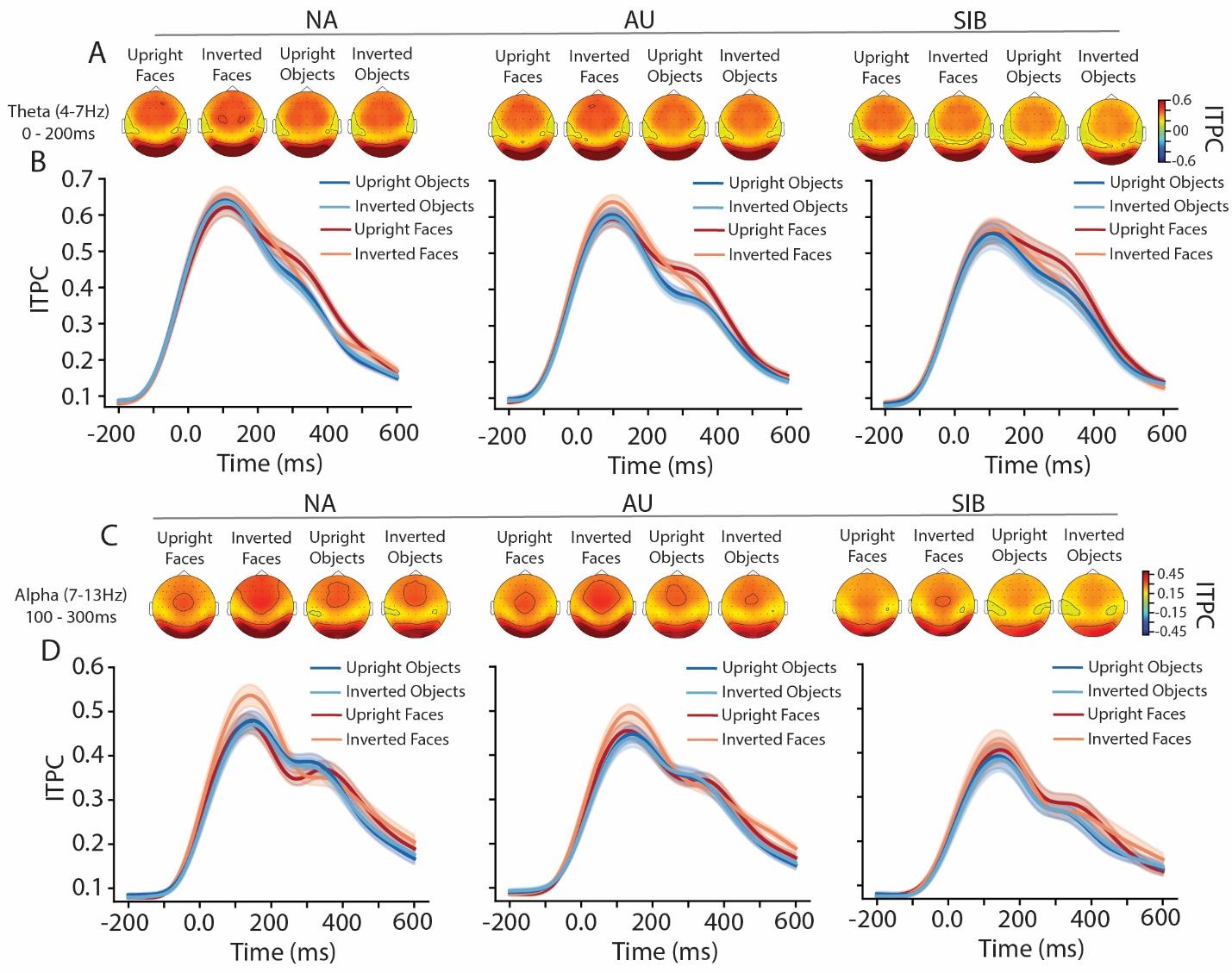


**Supplementary figure 4. Inter-trial phase coherence (ITPC) for theta and alpha band. (A)** Topographical representation of the ITPC for the theta band (4 – 7Hz) averaged between 0 to 200 ms in response to each type of stimulation and **(B)** time course of the ITPC averaged over a cluster of occipital channels in response to Upright Faces (red), Inverted Faces (orange), Upright Objects (blue) and Inverted Objects (light blue), for NA (left), AU (middle) and SIB (right). (**C**) and (**D**) same as (**A**) and (**B**) but for alpha (7 – 13Hz) activity.


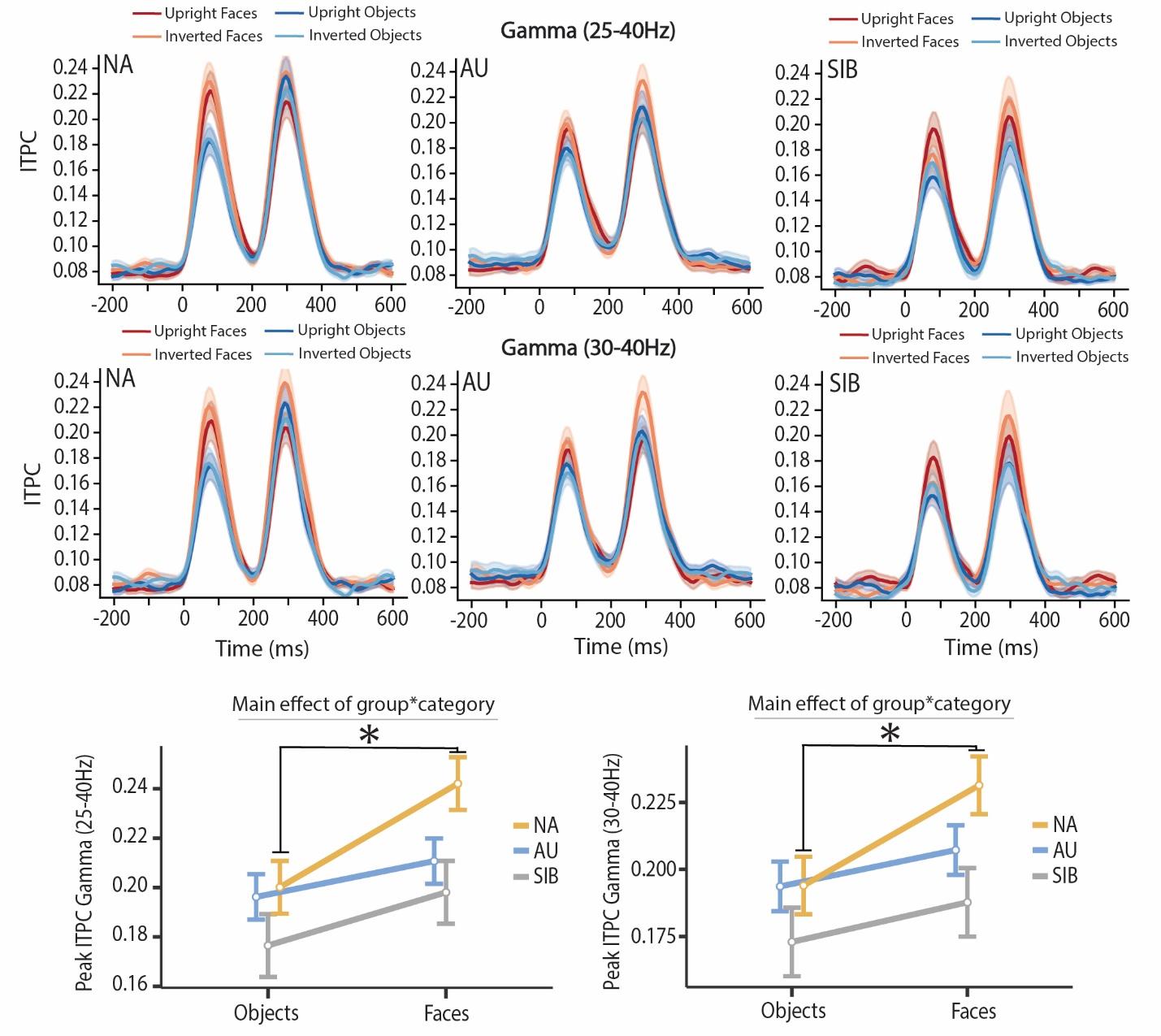


**Supplementary figure 5. Absence of face selectivity in gamma-band ITPC in the autistic group across two gamma frequency ranges.** Time course of the ITPC averaged over a cluster of occipital channels in response to Upright Faces (red), Inverted Faces (orange), Upright Objects (blue) and Inverted Objects (light blue), for NA (left), AU (middle) and SIB (right) groups. Results are displayed for the original low-gamma band (25–40 Hz; top) and for a more restricted gamma band (30–40 Hz; bottom). Significant main effects of the LMMs on the first peak (0-200ms) of gamma ITPC: Main effect of Category; interaction between Group and Category. (*) indicates significant post-hoc (α = 0.05 and Bonferroni condition).
